# Diffuse Large B Cell Lymphoma Cell Line U-2946: Model for *MCL1* Inhibitor Testing

**DOI:** 10.1371/journal.pone.0167599

**Published:** 2016-12-01

**Authors:** Hilmar Quentmeier, Hans G. Drexler, Vivien Hauer, Roderick A. F. MacLeod, Claudia Pommerenke, Cord C. Uphoff, Margarete Zaborski, Mattias Berglund, Gunilla Enblad, Rose-Marie Amini

**Affiliations:** 1 Department of Human and Animal Cell Lines, Leibniz-Institute DSMZ-German Collection of Microorganisms and Cell Cultures, Braunschweig, Germany; 2 Department of Immunology, Genetics and Pathology, Uppsala University and Uppsala University Hospital, Uppsala, Sweden; Universidad de Navarra, SPAIN

## Abstract

Diffuse large B cell lymphoma (DLBCL) is the most common form of non-Hodgkin lymphoma worldwide. We describe the establishment and molecular characteristics of the DLBCL cell line U-2946. This cell line was derived from a 52-year-old male with DLBCL. U-2946 cells carried the chromosomal translocation t(8;14) and strongly expressed *MYC*, but not the mature B-cell lymphoma associated oncogenes *BCL2* and *BCL6*. Instead, U-2946 cells expressed the antiapoptotic *BCL2* family member *MCL1* which was highly amplified genomically (14n). *MCL1* amplification is recurrent in DLBCL, especially in the activated B cell (ABC) variant. Results of microarray expression cluster analysis placed U-2946 together with ABC-, but apart from germinal center (GC)-type DLBCL cell lines. The 1q21.3 region including *MCL1* was focally coamplified with a short region of 17p11.2 (also present at 14n). The *MCL1* inhibitor A-1210477 triggered apoptosis in U-2946 (MCL1^pos^/BCL2^neg^) cells. In contrast to BCL2^pos^ DLBCL cell lines, U-2946 did not respond to the BCL2 inhibitor ABT-263. In conclusion, the novel characteristics of cell line U-2946 renders it a unique model system to test the function of small molecule inhibitors, especially when constructing a panel of DLBCL cell lines expressing broad combinations of antiapoptotic *BCL2*-family members.

## Introduction

Continuous cell lines have become indispensable tools in leukemia/lymphoma research. Self -renewing and transportable in the frozen state, cell lines provide unlimited supplies of cellular tumor material worldwide. Descendent from single cells of a patient´s tumor, they retain oncogenomic aberrations and gene expression patterns characteristic of the primary tumor. Tumor congruence is a key reason why cell lines are used as models to study the function of aberrantly expressed genes or to test the efficiency of novel anti-cancer drugs [1,2].

Diffuse large B cell lymphoma (DLBCL) is the most common form of lymphoma in adults. Various genes are recurrently affected by mutations and chromosomal aberrations therein, including the germinal center oncogenes *BCL2*, *BCL6* and *MYC* [3]. Expression array analysis has identified two molecularly distinct forms of the tumor, termed germinal center (GC) and activated B-cell (ABC) [4]. DLBCL-derived cell lines also show correspondingly distinct expression profiles allowing classification according to the GC- and ABC-scheme [5–9]. In contrast to GC-type DLBCL, ABC-type cells rely on the constitutive activation of the NF-kB pathway to block apoptosis [10]. Cell lines have been widely used to determine the effect of recurrent mutations or overexpressed genes on signaling pathways in ABC DLBCL and other lymphoma entities and to develop drugs for targeted therapies [5,7,10]. One important step in tumorigenesis is the loss of functional apoptosis, explaining why overexpression of antiapoptotic genes can contribute to tumorigenesis [11]. In DLBCL, the antiapoptotic genes *BCL2* and *MCL1* are recurrently overexpressed, as result of chromosomal translocations, amplification or other mechanisms [12–14]. We describe the establishment and molecular characteristics of the DLBCL-derived cell line U-2946. Due to an amplification on chr. 1q21.3, this cell line overexpresses *MCL1*. Several DLBCL cell lines have been used to validate novel drugs targeting antiapoptotic *BCL2* family members [13–18]. We propose U-2946 as auspicious model cell line which shows the rare combination of MCL1 positivity and BCL2 negativity.

## Materials and Methods

### Human cell lines

Authenticated stocks of cell line U-2946 were grown in RPMI 1640 (Invitrogen, Darmstadt, Germany) containing 10% fetal bovine serum (FBS) (Sigma-Aldrich, Taufkirchen, Germany). Cell lines applied in this study are all held by the DSMZ—German Collection of Microorganisms and Cell Cultures, Braunschweig, Germany (www.dsmz.de) or were supplied by the originators for research purpose. Detailed references and cultivation protocols have been described previously [19]. The *BCL2* family inhibitors ABT-263 and A-1210477 were obtained from Selleckchem (München, Germany).

### Cytogenetic analysis

Cells were harvested and fixed as described previously [20]. Spectral karyotyping (SKY) and fluorescence *in situ* hybridization (FISH) were performed as described previously [21]. Tilepath bacterial artificial chromosome (BAC) clones were sourced from BAC-PAC Resources (Children´s Hospital, Oakland, CA, USA), and SKY probes from Applied Spectral Imaging (Edingen, Germany). Probe selection was performed using the UCSC Genome Browser (https://genome-euro.ucsc.edu/) guided by the results of combined conventional cytogenetic, SKY and Cytoscan copy number array data (see below). For colocalization experiments commercial chromosome painting probes were mixed 1:1 with labelled BACs. Probe preparation and labelling were as described previously [21]. Briefly, BAC clone DNA was labelled by nick translation with dUTPs contrastingly labelled with fluors DY-495/547/590 purchased from Dyomics (Jena, Germany) and slide preparations counterstained with DAPI (4′,6-Diamidine-2′-phenylindole dihydrochloride) in Vector antifade mountant (Biozol, Eching, Germany). Imaging and analysis were performed using an Axioimager D1 microscope system equipped with an alpha-Plan Apochromat 100x objective (Zeiss, Goettingen, Germany) configured to a Spectral Imaging analysis system (Applied Spectral Imaging). For FISH, monochromatic fluor and DAPI signals were captured, merged and the pseudocolored images aligned automatically to generate reverse G-banding as described previously [21].

### Numerical aberrations

CytoScan HD Array (Affymetrix, Santa Clara, CA, USA) hybridization analysis was performed to identify numerical aberrations. DNA was prepared using the Qiagen Gentra Puregene Kit (Qiagen, Hilden, Germany). Data were analyzed using the Chromosome Analysis Suite software version 2.0.1.2 (Affymetrix).

### Cell surface marker analysis

Immunophenotyping was conducted on a FACSCalibur (Becton Dickinson, Heidelberg, Germany). Antibodies (Abs) against CD3 (Leu4), CD5 (UCHT2), CD10 (HI10a), CD19 (Leu12), CD20 (Leu16), CD34 (clone 581), CD37 (M-B371), CD38 (HIT2), CD138 (MI15) and HLA-DR (Tu39) were purchased from Becton Dickinson. Ab CD13 (My7) was from Beckman Coulter (Krefeld, Germany). Optimal dilutions of the Abs were determined with positive and negative control cell lines. FITC-labeled Abs against IgG, IgM, Ig kappa and Ig lambda were obtained from Southern Biotech/Biozol (Eching, Germany).

### Screening for immunoglobulin rearrangements and hypermutations

Rearrangement of the immunoglobulin (Ig) heavy chain was determined by genomic PCR using primers described by van Dongen et al. [22]. The PCR was performed for 35 cycles at 60°C annealing temperature. The level of Ig hypermutation was determined by sequencing the cloned PCR products. Ten clones were sequenced to screen for existence of U-2946 subclones.

### DNA microarray hybridization

Five hundred ng total RNA were used for biotin labelling according to the 3´ IVT Express Kit (Affymetrix); 7.5 μg of biotinylated cDNA were fragmented and placed in a hybridization cocktail containing four biotinylated hybridization controls (BioB, BioC, BioD, and Cre). Samples were hybridized to an identical lot of Affymetrix GeneChip HG-U133 Plus 2.0 for 16 h at 45°C. Steps for washing and SA-PE staining were processed on the fluidics station 450 using the recommended FS450 protocol (Affymetrix). Image analysis was performed on GCS3000 Scanner and GCOS1.2 Software Suite (Affymetrix). Data processing and analysis (probe summarisation, RMA-background correction and quantile normalization of spot intensities) were performed via R/Bioconductor using limma and affy packages [23–25].

### Quantitative real-time PCR analysis

Quantitative PCR was performed on a 7500 Applied Biosystems real-time PCR system (Darmstadt, Germany). RNA was prepared using the RNeasy Mini kit (Qiagen). Reverse transcription was performed using the SuperScript II reverse transcriptase kit (Invitrogen, Karlsruhe, Germany). TaqMan probes (Applied Biosystems) were used to quantify human *BCL2* (Hs00153350_m1), *BCL6* (Hs00153368_m1), *MAP2K3* (Hs00177127_m1), *MCL1* Exon 1/2 (Hs03043899_m1), *MYC* exon 1/2 (Hs00905030_m1) and *MYC* exon 2/3 (Hs00153408_m1) expression levels with *TBP* as endogenous control. Relative expression levels were calculated using the ΔΔCt method.

Ploidy levels of *BCL2*, *MAP2K3* and *MCL1* were determined by genomic PCR applying the SYBR green assay system (Applied Biosystems) with *ABL1* as internal control and NC-NC as diploid reference cell line. DNA was prepared using the High pure PCR template preparation kit (Roche Life Science, Mannheim, Germany). The applied primers are listed in S1 Table.

### Western blot analysis

Samples were prepared as described previously [26]. Anti BCL2 (1:500 dilution) and anti MCL1 Abs (1:250) were purchased from Becton Dickinson. Abs against the phosphorylated and unphosphorylated forms of p38 and MAP2K3 (1:10,000 each) and against BCL6 (1:1,000), BCLXL (1:1,000) and MYC (1:2,000) were obtained from Cell Signaling Technology (Leiden, The Netherlands), the GAPDH mouse monoclonal Ab (mAb) (1:5,000) was from Abcam (Cambridge, UK). Specific bands on nitrocellulose membranes were visualized with the biotin/streptavidin-horseradish peroxidase system (GE Healthcare, Little Chalfont, UK) in combination with the “Renaissance Western Blot Chemoluminescence Reagent” protocol (Perkin Elmer, Waltham, MA, USA).

### 3H-thymidine uptake

Assays of 3H-thymidine incorporation were executed as follows: 1.25 x 10^4^ cells (in 100 μl) were seeded in triplicate in 96-well flat-bottom microtiter cell culture plates. Inhibitors were added as 2x concentrated solution in a 100 μl volume. For the last 3 h of the incubation period, 1 μCi 3H-thymidine (Hartmann Analytic, Braunschweig, Germany) was added to each well.

### Detection of apoptotic cells

Apoptotic cells were detected and quantified with the annexin-V/propidium iodide method using the TACS Annexin-V-FITC kit (R&D Systems, Wiesbaden, Germany) according to the manufacturer’s instructions. Binding of fluorescein isothiocyanate-labeled annexin-V and propidium iodide staining of the cells were determined by flow cytometry on the FACSCalibur (Becton Dickinson).

## Results

### Clinical history

The patient was a previously healthy man, age 52 years who sought medical care due to abdominal pain and tiredness. Computer tomography examination revealed an abdominal tumor. In July 1996 he underwent a right-sided hemicolectomy due to a tumor of the ascending colon. The tumor, 15 cm in diameter, removed macroscopically radically. Pathological examination showed DLBCL. Diagnostic work-up revealed lymphomatous engagement in the right testicle and in paratesticular tissue with a diameter of >10 cm, in lymph nodes and in the bone marrow (stage IVB). International Prognostic Index was retrospectively assigned to 3 (Stage IV, >1 extranodal site and elevated S-LDH (7 x upper normal limit)). At diagnosis he also had a palsy of the left N. abducens but examination of cerebrospinal fluid showed no signs of lymphoma and a CT of the brain was normal.

He was treated with VACOP-B [27]: a weekly schedule of alternating chemotherapy routinely used for aggressive lymphomas at our department at that time. He also received intrathecal injections of methotrexate 12.5 mg. At week 7 he had progressive disease and the treatment was changed to MIME [28], which was given for two courses before the patient progressed and received palliative radiotherapy. His disease progressed rapidly and he developed pleural effusion and leukemic lymphoma with a WBC count of > 100 x 10^9^/l. The patient gave informed consent to use the pleural effusion for research including the possible establishment of a cell line. He died of progressive lymphoma three months after diagnosis. The overall picture of the lymphoma was an extremely aggressive disease with fast progression and very limited sensitivity to chemotherapy.

Large blast cells with the appearance of both immunoblasts and centroblasts grew in a diffuse and infiltrative pattern in the intestine. Several mitotic figures and apoptotic cells were present. Immunohistochemical stainings were performed and the lymphoma cells were positive for CD20, CD79a, CD10, BCL-6, MYC, p53 and negative for BCL-2, CD5, CD30, cyclin D1, SOX11 with a high proliferation index of Ki67. Partial expression of MUM1 and FOXP1 was detected. According to the WHO classification and update [29,30] the lymphoma would be classified as a DLBCL with a germinal-center derived phenotype according to the Hans et al algorithm (S1 Fig) [31,32]. In addition, *MYC* translocation was detected by in situ hybridization.

### Cell culture

Tumor cells from pleural effusion were obtained by Ficoll-Isopaque (Lymphoprep, Nycomed) gradient separation and cultured in RPMI 1640 containing 10% of FBS, glutamine and antibiotics (100 IU/ml penicillin and 50 μg/ml streptomycin) at 37°C in 5% CO_2_ humidified environment. Cells were split every 72 h with an optimal density of 0.3 x 10^5^ cells/ml and a doubling time of 48 h. After one month, the cell line U-2946 was considered established and grew in continuous culture in RPMI 1640 with 10% FBS for another 2 months. U-2946 cells tested EBV-negative excluding a lymphoblastoid origin. May-Grünwald-Giemsa stain of the cell line is shown in S1 Fig.

### U-2946: immunoglobulin rearrangement and immunophenotype

We tested the Ig rearrangement status of cell line U-2946 using previously described primers [22]. The cell line was found to carry an IGHV4-59 rearrangement with 6% hypermutation. Consistent with the patient´s tumor, U-2946 cells showed the typical immunophenotype of a B-lymphoma cell line (Table 1, S2 Fig).

**Table 1:** Immunoprofile of patient and–derived cell line U-2946.

	patient	U-2946		patient	U-2946
CD3	neg	neg	CD37	n.d.	pos
CD5	neg	neg	CD38	n.d.	pos
CD10	pos	pos	CD138	n.d.	neg
CD13	n.d.	neg	HLA-DR	n.d.	pos
CD19	pos	pos	IgG	n.d.	neg
CD20	pos	pos	IgM	n.d.	pos
CD22	pos	n.d.	kappa	n.d.	neg
CD34	n.d.	neg	lambda	n.d.	pos

Expression of surface markers was assessed by flow cytometry with the appropriate isotype controls. pos, ≥ 15% express antigen

neg, < 15% express antigen; n.d., not done.

### Karyotyping

**Fig 1:**
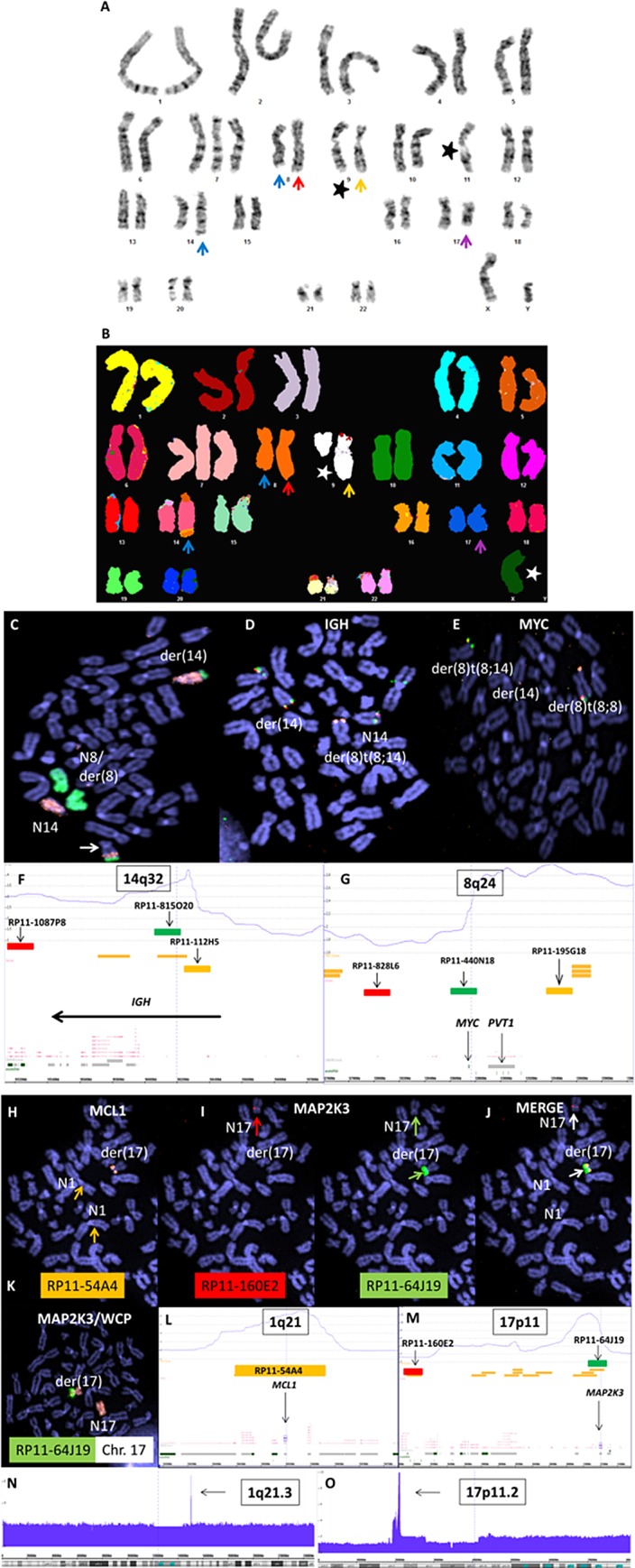
Cytogenetic analysis of U-2946 cells. A) Classical and B) Spectral Karyotyping shows key oncogenomic rearrangements present in U-2946 cells. BAC clones used for FISH are shown along with corresponding genomic copy number plots. Blue arrows show t(8;14)(q24;q32), red arrows der(8)t(8;8)(p22;q24), yellow arrows der(9)t(2;9) and purple arrows add(17)(p11). Note differences between cells in A and B reflecting clonal heterogeneity indicated by stars. C-G) Image depicts analysis of t(8;14) by chromosome painting (C), and single locus FISH using probes for IGH (D) and MYC (E). Positions of BAC clones are shown for IGH (F) and MYC loci (G). Note partial duplication of the terminal der(14) long arm region including IGH/MYC (arrow) present in a subclone (C). H-M) Analysis of MCL1/MAP2K3 coamplification: gold signals from MCL1 clone (RP11-54A4), barely visible at native loci (arrows), are intense on der(17) due to amplification (H). Similarly, green signals from 17p11 clone (RP11-64J19) covering MAP2K3 are amplified, while red signals (RP11-160E2) from the neigboring hemizygously deleted region are present on the unrearranged chromosome only. Chromosome painting shows localization of amplicon to 17p (K). Genomic copy number plots show consistency with FISH amplification data for both MCL1 (L) and MAP2K3 (M). N-O) Cytoscan HD array analysis revealed prominent amplification of 1q21.3 (N) and 17p11.2 (O).

The consensus karyotype, performed using classical (Fig 1A) and spectral karyotyping (Fig 1B), together with FISH covering the *IGH* and *MYC* loci (Fig 1C–1G) was resolved to: 47<2n>XY, +7, der(9)t(2;9)(p25;p21), der(8)inv(8)(p22;q24), der(9)t(2;9)(p25;p22), add(17)(p11). FISH revealed canonical juxtaposition of *IGH* and *MYC*, typical of B-cell neoplasms plus an additional non *IGH* rearrangement, der(8)t(8;8)(p23;q24), effecting copy number increase of the terminal 8q region just including *PVT1*, but excluding *MYC*.

Cytogenetic analysis also revealed a novel rearrangement, focal co-amplification of 1q21 and 17p11.2 replacing a deleted segment of chromosome 17p (Fig 1H–1M). The ca. 67 kbp–amplicon at 1q21 covered two loci (*MCL1*, *ENSA*), while that at 1p11 extending some 461 kbp covered four loci (*USP22*, *DHRS7B*, *TMEM11*, *MAP2K3*). Coamplification of gene pairs normally arises when a genomically remote gene pair, ectopically juxtaposed or fused by chromosome rearrangement is locally subjected to linear copy number amplification, e.g. following selection. Such copy number aberrations are hallmarks of oncogenes and are thought to arise by conferral of proliferation or antiapoptopic advantage.

### U-2946 with ABC marker expression

**Fig 2:**
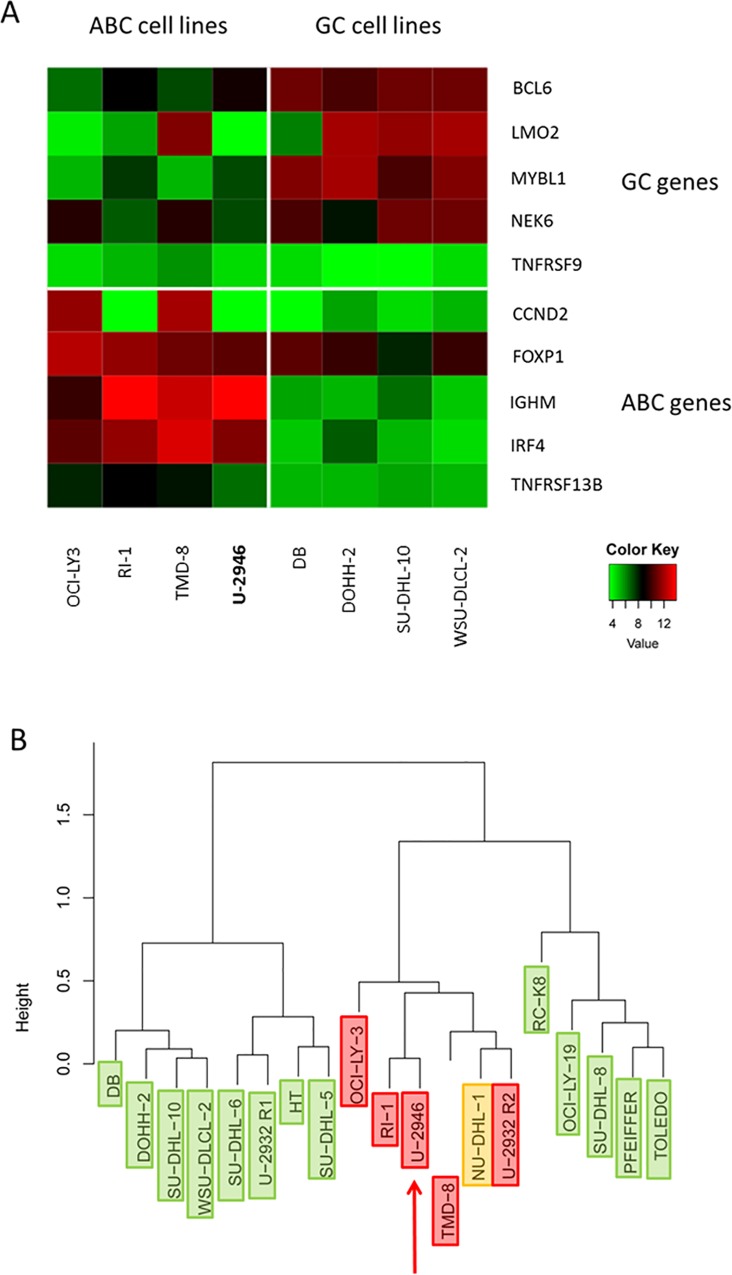
U-2946: an ABC DLBCL cell line. A) heatmap of ABC/GC gene expression, data from expression array analysis. The majority of ten ABC and GC markers described by Ruminy et al. [33] class U-2946 as ABC DLBCL cell line. B) Applying the same set of marker genes, cell line U-2946 clustered together with described ABC DLBCL cell lines (red), not with GC cell lines (green) [5–9]. Cell line NU-DHL-1 (orange) was discordantly categorized by different authors [6,8]. We showed that subclone R1 of the ABC cell line U-2932 highly expressed various GC markers, absent from subclone R2 [34].

Expression array analysis allows distinction between two forms of DLBCL, the GC and the ABC forms [4]. Based on five ABC marker genes and five GC markers [33], cell line U-2946 was assigned to the ABC group (Fig 2A). Unsupervised clustering of 19 DLBCL cell lines confirmed the ABC character of this cell line (Fig 2B).

### *BCL2*, *BCL6* and *MYC*

**Fig 3:**
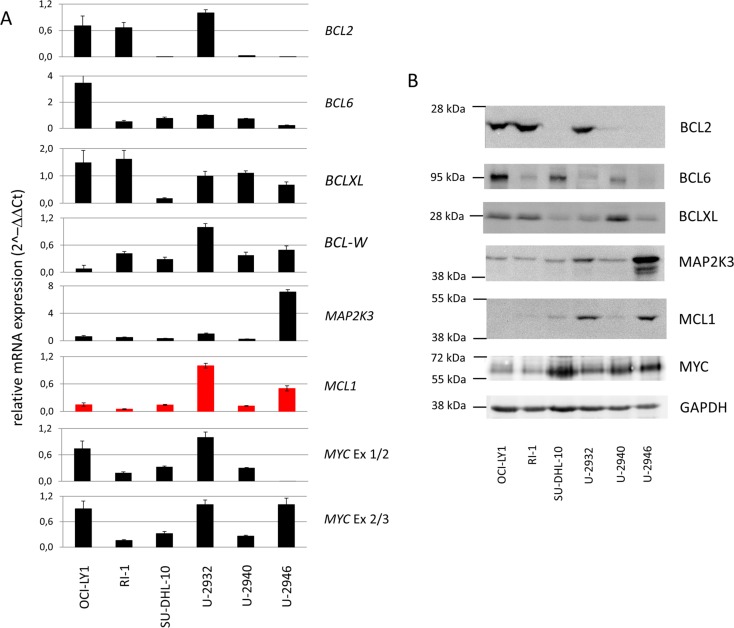
Expression of BCL2, BCL6, BCLXL, MAP2K3, MCL1, and MYC in DLBCL cell lines. A) qRT-PCR and B) Western blot analysis was performed to assess the expression of oncogenes in U-2946 and other DLBCL cell lines. The bars in A) indicate means with standard deviation (n = 3). The ct values for BCL-W in qRT-PCR were high (>30) in all cell lines tested explaining absence of specific bands in Western blot analysis (not shown). Note the high-level expression of MCL1 (red) in cell lines U-2946 and U-2932.

Mature B cell lymphomas often carry rearrangements and numerical aberrations leading to aberrant expression of *BCL2*, *BCL6* and *MYC*. These aberrations can occur in the same tumors, which are then called “double-hit” or “triple-hit” lymphoma according to their aberration scores. Cell lines representing double-hit and triple-hit lymphomas have recently been reviewed in detail [35]. Amplifications or canonical translocations affecting chromosomes 18q21 (*BCL2*), 3q27 (*BCL6*) were not detected in U-2946 cells. As shown in Fig 1C–1G, *MYC* on 8q24 was fused to *IGH* on 14q32. In accordance with the genomic findings, cell line U-2946 was BCL2^neg^/BCL6^low^/MYC^pos^ (Fig 3A and 3B). 5´RACE revealed that the *MYC* transcript began in intron 1/2, not in exon 1 (data not shown). Accordingly qRT-PCR showed *MYC* transcript in exon 2 but not in exon 1 suggesting (i) that the genomic break was located in intron 1/2, and (ii) that translation started with the alternative ATG start codon in exon 2 and not with the “internal ribosome entry site” (IRES)-mediated CTG codon in exon 1 (Fig 3A).

### Numerical aberrations are responsible for aberrant gene expression in U-2946

To find genes conspicuously expressed in U-2946, we compared expression array data of this cell line with those of 55 other certified B-lymphoma cell lines spanning the whole spectrum of B-NHL. Thirty-nine of the 46 marker genes (85%) were upregulated in U-2946; another 7 genes (15%) lay below the average of B-lymphoma cell lines (S3 Fig). High density genomic array analyses revealed that 6/7 underexpressed genes mapped to haploid regions (S2 Table). Five genes were amplified, all of them overexpressed (S2 Table). Thus, we found an almost perfect correlation between deletion and lack of expression on the one side, and amplification and overexpression on the other. The most prominent amplifications were at regions 1q21.3 (6n) (Fig 1N) and 17p11.2 (4n-10n) (Fig 1O). The amplified genes are listed in S3 Table.

### *MAP2K3*: amplified but inactive

**Fig 4:**
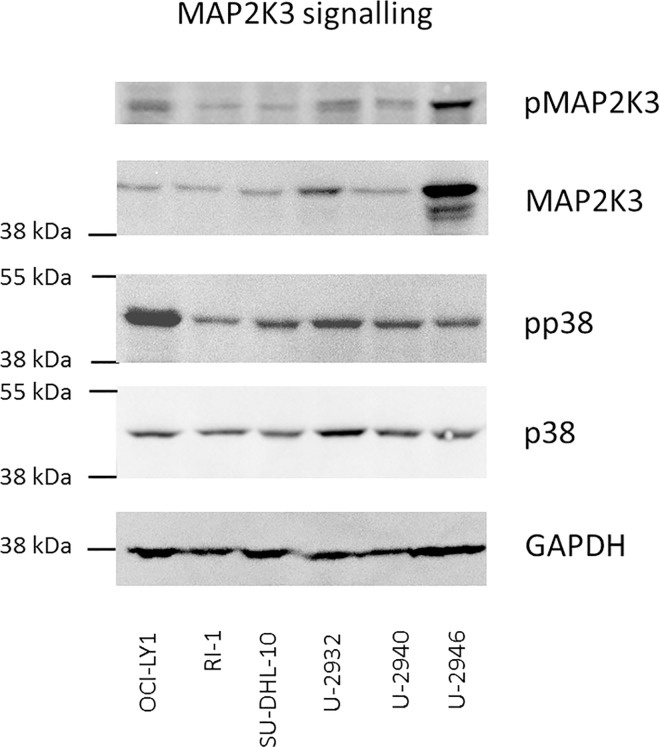
Phosphorylation of the MAP2K3/p38 pathway. Expression and phosphorylation of MAP2K3 and p38 in U-2946 and five other DLBCL cell lines was determined by Western blot analysis. Note that overexpression of MAP2K3 was not accompanied by high phosphorylation of the MAP2K3 target in cell line U-2946.

Located on 17p11.2, *MAP2K3* (*MEK3*) was amplified in cell line U-2946 (Fig 1O, S3 Table). Amplification of the gene was accompanied by overexpression of *MAP2K3*, at the mRNA and protein levels (Fig 3A and 3B). This kinase is known to activate the p38 MAPK pathway [36]. Sequencing of *MAP2K3* cDNA showed that the gene was not mutated. Nevertheless, cell lines with low expression of *MAP2K3* showed the same p38 MAPK phosphorylation level as cell line U-2946 (Fig 4). Thus, *MAP2K3* was highly expressed in cell line U-2946, but did not aberrantly trigger the p38 MAPK pathway.

### *MCL1* amplified on der chr17

Like *BCL2*, *MCL1* is an antiapoptotic gene and recurrently overexpressed in DLBCL [13,14]. Accordingly, the 1q21 region including the *MCL1* locus is recurrently amplified in DLBCL [37]. A positive correlation of *MCL1* amplification and gene expression was specifically observed in ABC DLBCL [14]. Thus 1q21.3 amplification (including the *MCL1* gene locus) is consistent with the ABC character proposed here for cell line U-2946 (Fig 1N, S3 Table). FISH analysis revealed that *MCL1* was translocated to chr17, specifically to the amplified region on 17p11.2 including *MAP2K3* (Fig 1H–1M).

To elucidate whether *MCL1* amplification might correlate with the gene´s overexpression, we performed qRT-PCR and Western blot analysis for U-2946 in comparison with other DLBCL cell lines (Fig 3A and 3B). Cell line U-2946 was MCL1^pos^/BCL2^neg^. All other cell lines showed different combinations of *MCL1* and *BCL2* expression (y3A and 3B). Overexpression of both genes was always accompanied by genomic amplification, except in OCI-LY1, where we observed *BCL2* mRNA overexpression without genomic amplification (S4 Fig). This cell line is known to carry the t(14;18) effecting *IGH/BCL2* juxtaposition, another well-described cause of aberrant *BCL2* expression [12,38].

### *MCL1* with antiapoptotic function in cell line U-2946

**Fig 5:**
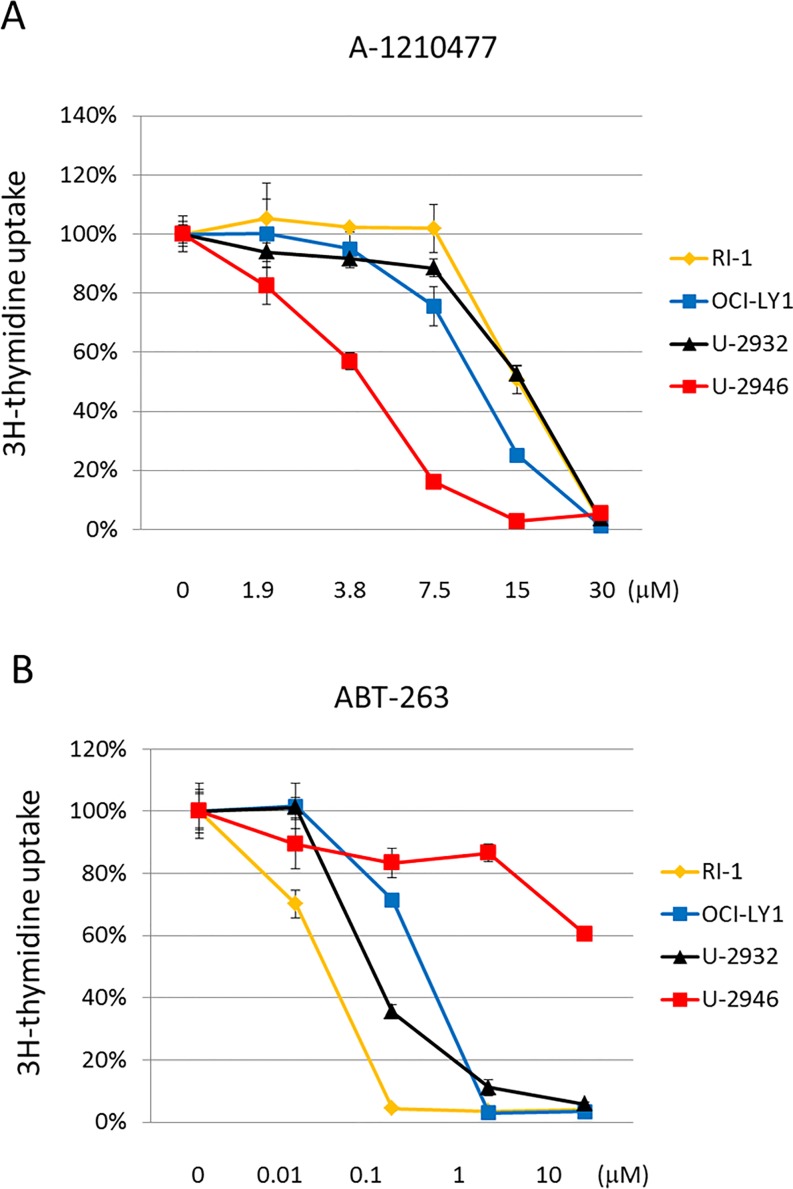
Inhibition of BCL2 and MCL1: effect on proliferation. The effect of A) A-1210477 (MCL1 inhibitor) and B) ABT-263 (BCL2 inhibitor) on proliferation of DLBCL cell lines was assessed by 3H-thymidine uptake (d2). Cell lines OCI-LY1 and RI-1 are MCL1^neg^/BCL2^pos^, cell line U-2932 is MCL1^pos^/BCL2^pos^ and cell line U-2946 is MCL1^pos^/BCL2^neg^. Especially note the significant reduction of 3H-thymidine incorporation in cell line U-2946 after addition of the MCL1 inhibitor.

**Fig 6:**
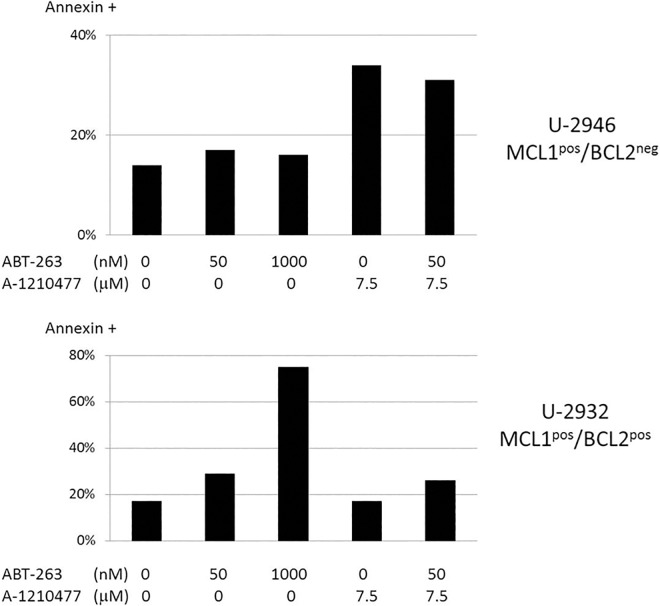
Inhibition of BCL2 and MCL1: effect on apoptosis. Apoptosis was assessed by Annexin-V staining, 24 h after addition of a BCL2 inhibitor (ABT-263) or an MCL1 inhibitor (A-1210477). The MCL1 inhibitor triggered apoptosis in the MCL1^pos^/BCL2^neg^ cell line U-2946, BCL2 inhibition induced apoptosis in the MCL1^pos^/BCL2^pos^ cell line U-2932. Although expressing MCL1, cell line U-2932 was not responsive to MCL1 inhibition, suggesting dominant function of BCL2. The data were reproduced in two independent experiments.

To find out whether *MCL1* and *BCL2* were critical for cell survival in our DLBCL cell lines, we incubated the cell lines with inhibitors of *MCL1* (A-1210477) or *BCL2*, *BCL-XL* and *BCL-W* (ABT-263). The *MCL1* inhibitor (A-1210477, 7.5 μM) substantially reduced 3H-thymidine uptake and triggered apoptosis in the MCL1^pos^ cell line U-2946, while MCL1^neg^ cell lines were not affected (Figs 5A and 6). All BCL2^pos^ cell lines (OCI-LY1, RI-1, U-2932) were responsive to low doses of the BCL2 inhibitor (Figs 5B and 6). Noteworthingly, the pharmacological MCL1 inhibitor left the MCL1^pos^/BCL2^pos^ cell line U-2932 unperturbed. Even in the presence of low concentrations of the *BCL2* inhibitor (50 nM ABT-263), the *MCL1* inhibitor (7.5 μM A-1219477) did not have any effect on U-2932 cells, suggesting that *BCL2* was dominant over *MCL1* in the U-2932 cell system (Fig 6 and S5 Fig).

## Discussion

Cell line U-2946 was established in 1996 from a 52-year-old male patient with terminal DLBCL following standard chemotherapy. Immunohistochemical staining classified the tumor into a GC-derived phenotype according to the Hans classification [39]. However, the only data published so far on cell line U-2946 describe the strong expression of *ZAP70* and *PKC-ϐ II*, a feature occurring in the more aggressive clinical form of *non*-GC DLBCL [39]. Expression profiling confirmed that *ZAP70* levels were much higher in U-2946 than in the average of 56 B-lymphoma cell lines tested. Also consistent with the non-GC character of this cell line, U-2946 clustered together with other ABC DLBCL cell lines, as evidenced by gene expression analysis, applying a short-list of five ABC marker genes and five GC markers.

Translocations and/or numerical aberrations affecting the germinal center oncogenes *BCL2* and *BCL6* were not detected in U-2946 cells. FISH revealed the canonical juxtaposition of *IGH* and *MYC*, leading to overexpression of the oncogene in this cell line. The most prominent numerical abnormalities in U-2946 were amplifications on chromosomes 1q21.3 and 17p11.2. Both sites were coamplified on a derivative chromosome 17. *MAP2K3*, on 17p21.3, and *MCL1*, located on 1q21.3, were highly expressed. The *MAP2K3* overexpression did not lead to unusual phosphorylation of the downstream target p38 MAPK, suggesting that amplification of the *MAP2K3* was a passenger or helper mutation and that amplification of *MCL1* might be the true oncogenic event. *MCL1* is frequently expressed in DLBCL, in the ABC subtype more often than in GC and also at higher expression levels, which correlates with higher frequency of 1q21 amplification in ABC cases [13,14]. *MCL1* rescues cells from apoptosis by binding and sequestering proapoptotic *BCL2*-family members. Small-molecule inhibitors, developed to trigger apoptosis, have been tested in cell lines expressing antiapoptotic *BCL2*-family proteins [13,15–18]. Recently, it has been shown that DLBCL can be divided into BCL2-dependent and MCL1-dependent groups [40]. Cell line U-2946 expresses *MCL1*, but not *BCL2* and only low levels of *BCL-XL*. Accordingly, apoptosis was induced by the *MCL1* inhibitor A-1210477, while the *BCL2/BCL-XL* inhibitor ABT-263 (Navitoclax) had no effect. Other DLBCL cell lines expressed *BCL2* and responded to ABT-263. U-2932 was one of these BCL2^pos^ cell lines. This cell line also expressed MCL1, but did not respond to the MCL1 inhibitor. Even with low doses of the BCL2 inhibitor, A-1210477 was inefficient which suggests that the effect of BCL2 is dominant over MCL1, at least in this cell system.

We conclude that the MCL1^pos^/BCL2^neg^ cell line U-2946 promises to be a valuable model for inhibitor studies, useful especially in combination with cell lines expressing other antiapoptotic *BCL2* family members. Cell line U-2946 will be available from the public repository DSMZ (www.dsmz.de).

## Conclusions

We describe the establishment and characteristics of the DLBCL cell line U-2946. According to gene expression data, the cell line clusters together with ABC DLBCL cell lines. The cell line carries an amplification of the antiapoptotic *BCL2* family member *MCL1*. The *MCL1* chromosomal region (1q21.3) was involved in a translocation to chromosome 17. The amplified *MCL1* gene is highly expressed in the cell line (MCL1^pos^/BCL2^neg^). Inhibitor experiments revealed the importance of *MCL1* for growth and survival of U-2946 cells. Cell line U-2946 will prove to be an important model system for the functional analysis of small BCL2-family inhibitors, especially in a panel of certified DLBCL cell lines showing other *MCL1* and *BCL2* expression profiles.

## Supporting Information

S1 Fig Stainings of lymphoma cells and cell line. A-E) Immunohistochemical stainings of lymphoma cells. Positive: BCL6 (A), CD10 (B), CD20 (C), CD79a, FOXP1, MUM1 (partial), MYC (D), p53 and negative: BCL2 (E), CD3, CD5, CD30, cyclin D1. (F) May-Grünwald-Giemsa stain of cell line U-2946.
(TIF)

S2 Fig Immunophenotype of cell line U-2946. Cells were stained with Abs against the antigens indicated and analyzed by flow cytometry.
(TIF)

S3 Fig U-2946 vs 55 B-lymphoma cell lines–the top differentially expressed genes. The 47 genes with strongest expression differences between U-2946 and median of 55 B-lymphoma cell lines in relation to variance (IQR, interquartile range)–highest differences on the left. Data base on expression array analyses. Red dots, cell line U-2946.
(TIF)

S4 Fig Ploidy and gene expression. Quantitative genomic PCR (upper) and qRT-PCR (lower) detecting correlation between amplification and RNA expression in *BCL2*, *MAP2K3* and *MCL1*. The bars indicate means with standard deviation (n = 3). NC-NC as diploid reference cell line for genomic PCR.
(TIF)

S5 Fig *MCL1* and *BCL2* inhibition. 3H-thymidine uptake after 48 h. The *MCL1* inhibitor A-1210477 (7.5 μM) inhibits growth of the MCL1^pos^/BCL2^neg^ cell line U-2946, but has no effect on the MCL1^pos^/BCL2^pos^ cell line U-2932 –neither alone nor together with suboptimal doses of the *BCL2* inhibitor ABT-263 (50 nM). The bars indicate means with standard deviation (n = 3).
(TIF)

S1 Table Primers for genomic PCR. (XLSX)

S2 Table 46 top outliers in U-2946 vs. 55 B-lymphoma cell lines. Outliers are listed according to chromosomal position. Note the perfect correlation between genomic imbalances and expression in cell line U-2946, 6/6 hemiploid genes (black and bold) being repressed, 5/5 amplified genes (red and bold) being overexpressed. Up, higher than average; down, lower than average.
(XLSX)

S3 Table Amplified genes in cell line U-2946. Amplified genes on 1q21.3 are listed from centromere to telomere, genes on 17p11.2 from telomere to centromere. Bold: strongly expressed genes as assessed by expression array analysis.
(XLSX)
