# Supplementary figures and images for: Diffuse Large B Cell Lymphoma Cell Line U-2946: Model for MCL1 Inhibitor Testing
Source: PLoS One. 2016 Dec 1;11(12):e0167599. doi: 10.1371/journal.pone.0167599 (PMC5132233; doi:10.1371/journal.pone.0167599)

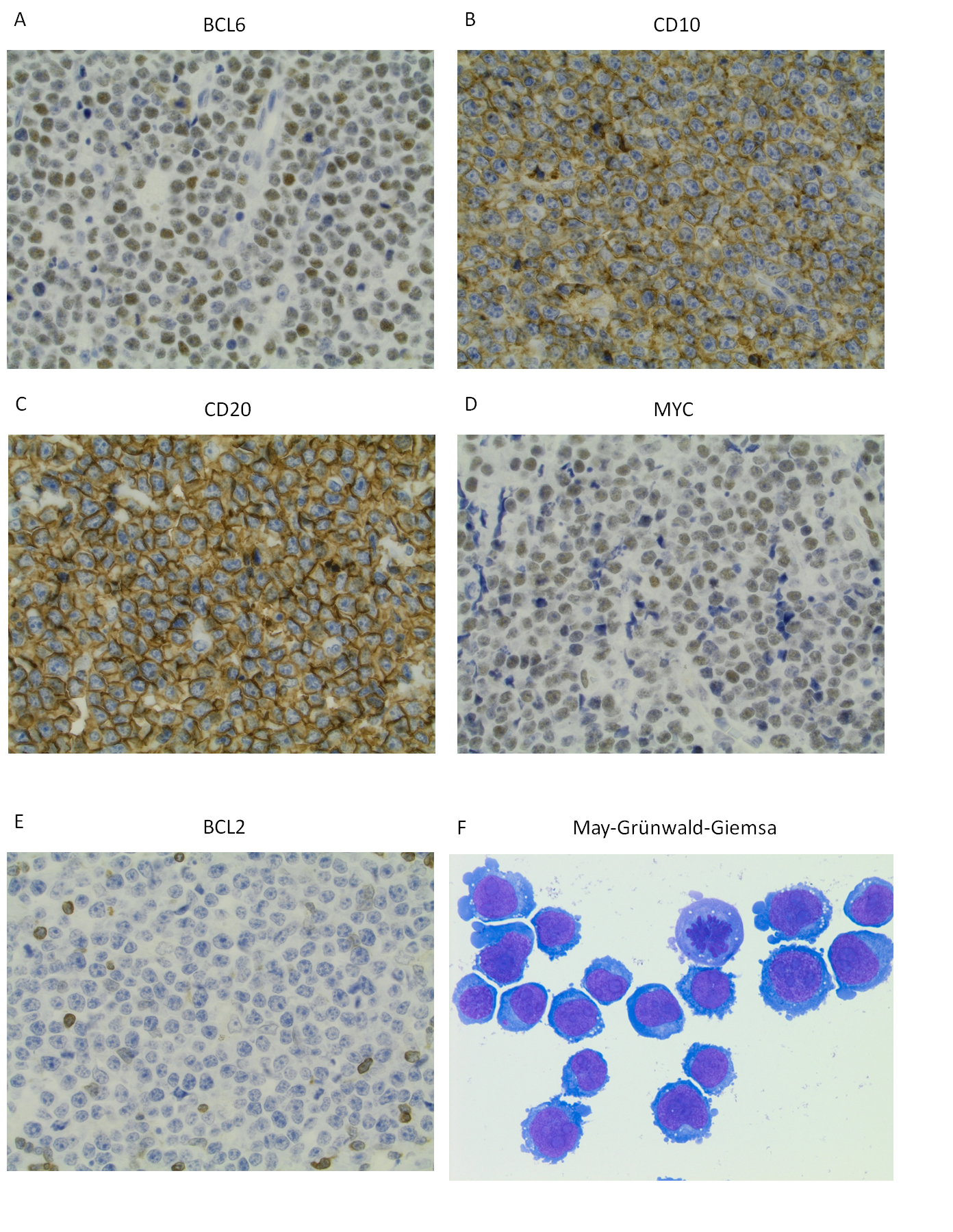

Supplement: S1 Fig — A-E) Immunohistochemical stainings of lymphoma cells. Positive: BCL6 (A), CD10 (B), CD20 (C), CD79a, FOXP1, MUM1 (partial), MYC (D), p53 and negative: BCL2 (E), CD3, CD5, CD30, cyclin D1. (F) May-Grünwald-Giemsa stain of cell line U-2946. (TIF) [file pone.0167599.s001.tif]

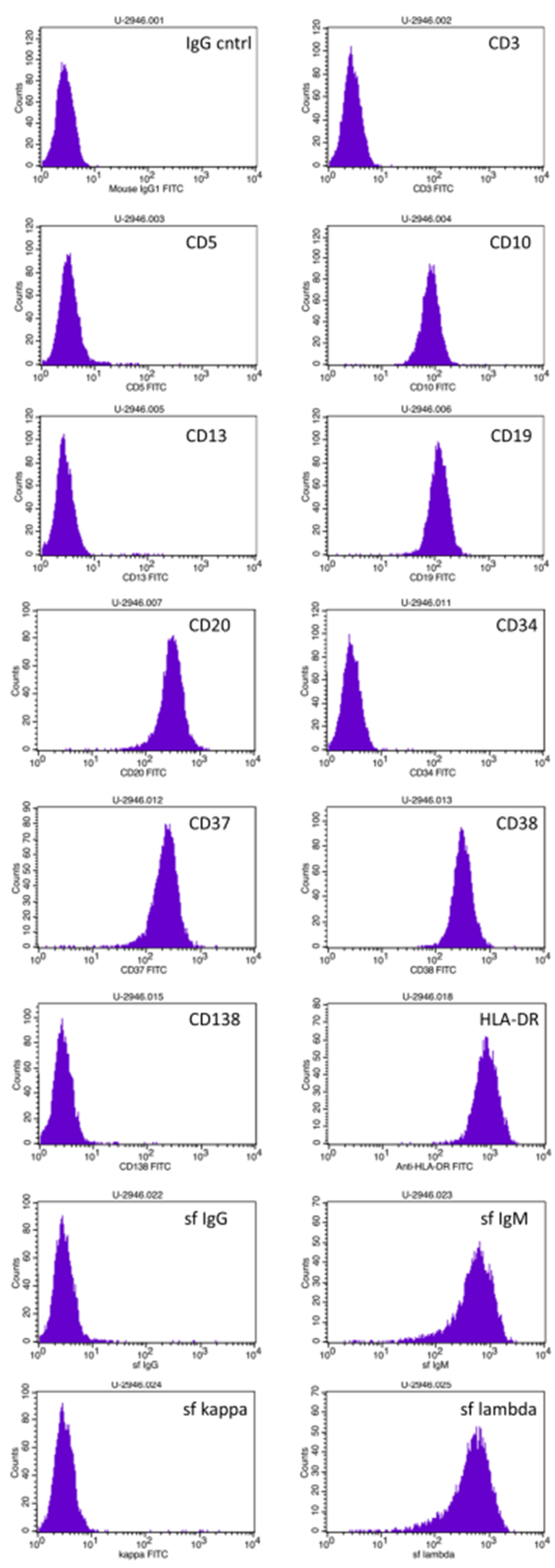

Supplement: S2 Fig — Cells were stained with Abs against the antigens indicated and analyzed by flow cytometry. (TIF) [file pone.0167599.s002.tif]

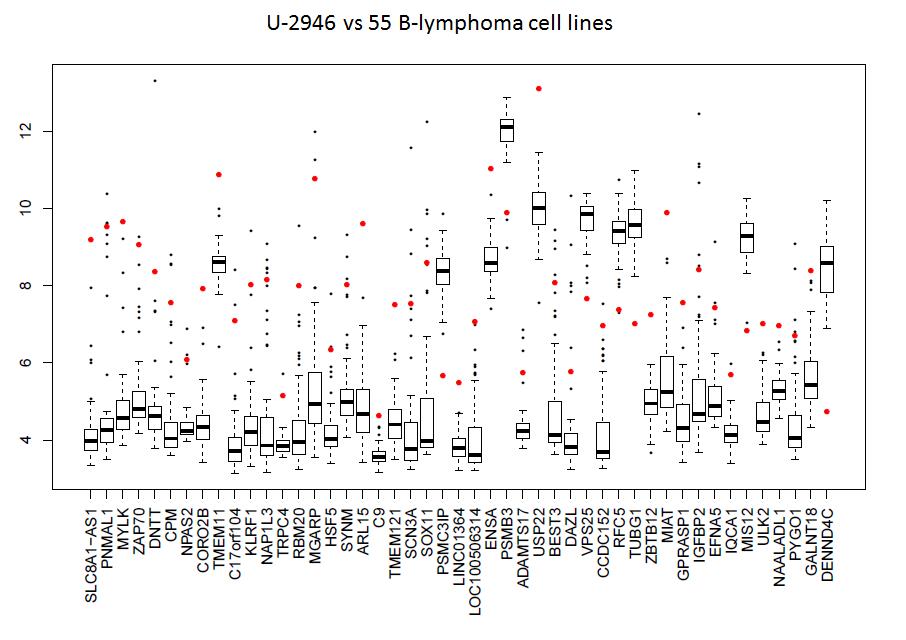

Supplement: S3 Fig — The 47 genes with strongest expression differences between U-2946 and median of 55 B-lymphoma cell lines in relation to variance (IQR, interquartile range)–highest differences on the left. Data base on expression array analyses. Red dots, cell line U-2946. (TIF) [file pone.0167599.s003.tif]

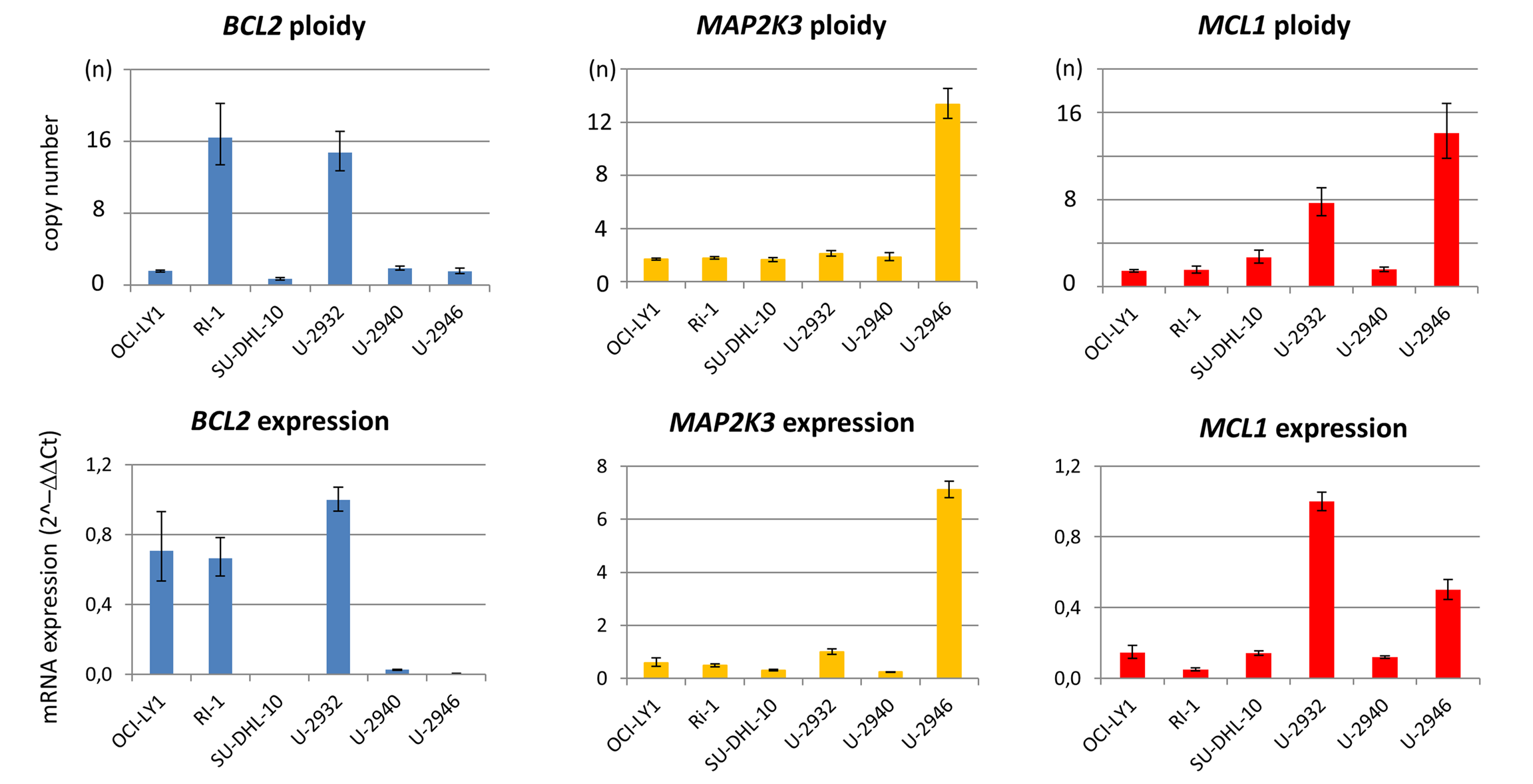

Supplement: S4 Fig — Quantitative genomic PCR (upper) and qRT-PCR (lower) detecting correlation between amplification and RNA expression in BCL2, MAP2K3 and MCL1. The bars indicate means with standard deviation (n = 3). NC-NC as diploid reference cell line for genomic PCR. (TIF) [file pone.0167599.s004.tif]

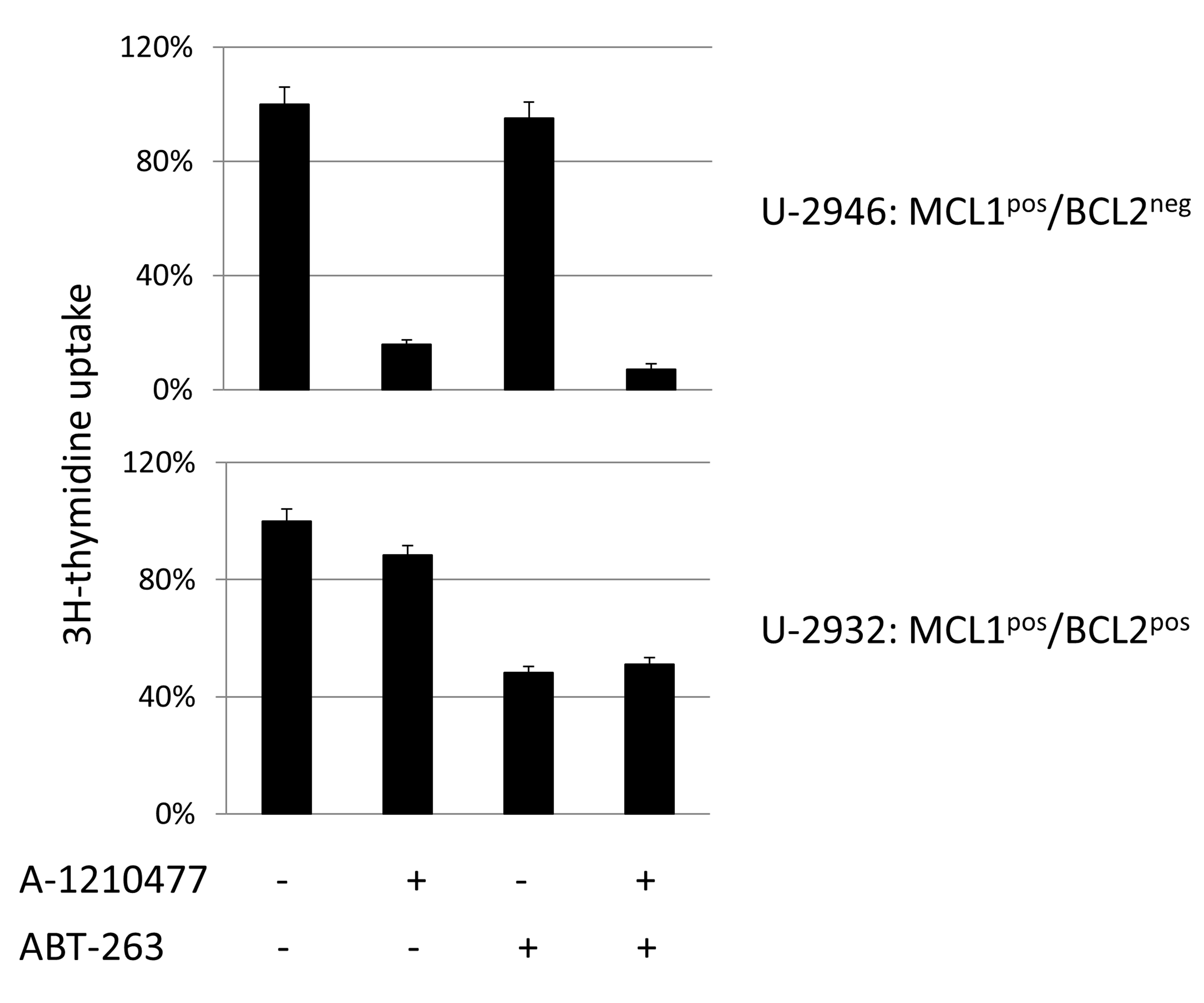

Supplement: S5 Fig — 3H-thymidine uptake after 48 h. The MCL1 inhibitor A-1210477 (7.5 μM) inhibits growth of the MCL1pos/BCL2neg cell line U-2946, but has no effect on the MCL1pos/BCL2pos cell line U-2932 –neither alone nor together with suboptimal doses of the BCL2 inhibitor ABT-263 (50 nM). The bars indicate means with standard deviation (n = 3). (TIF) [file pone.0167599.s005.tif]
